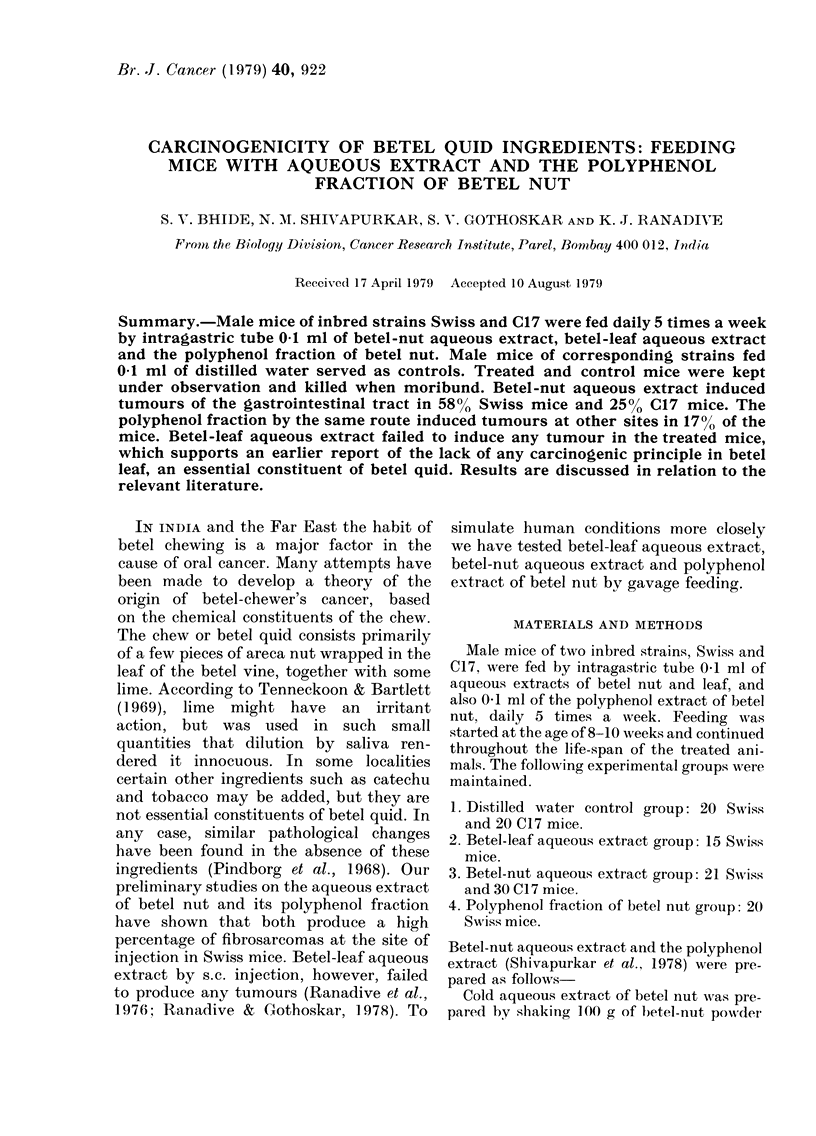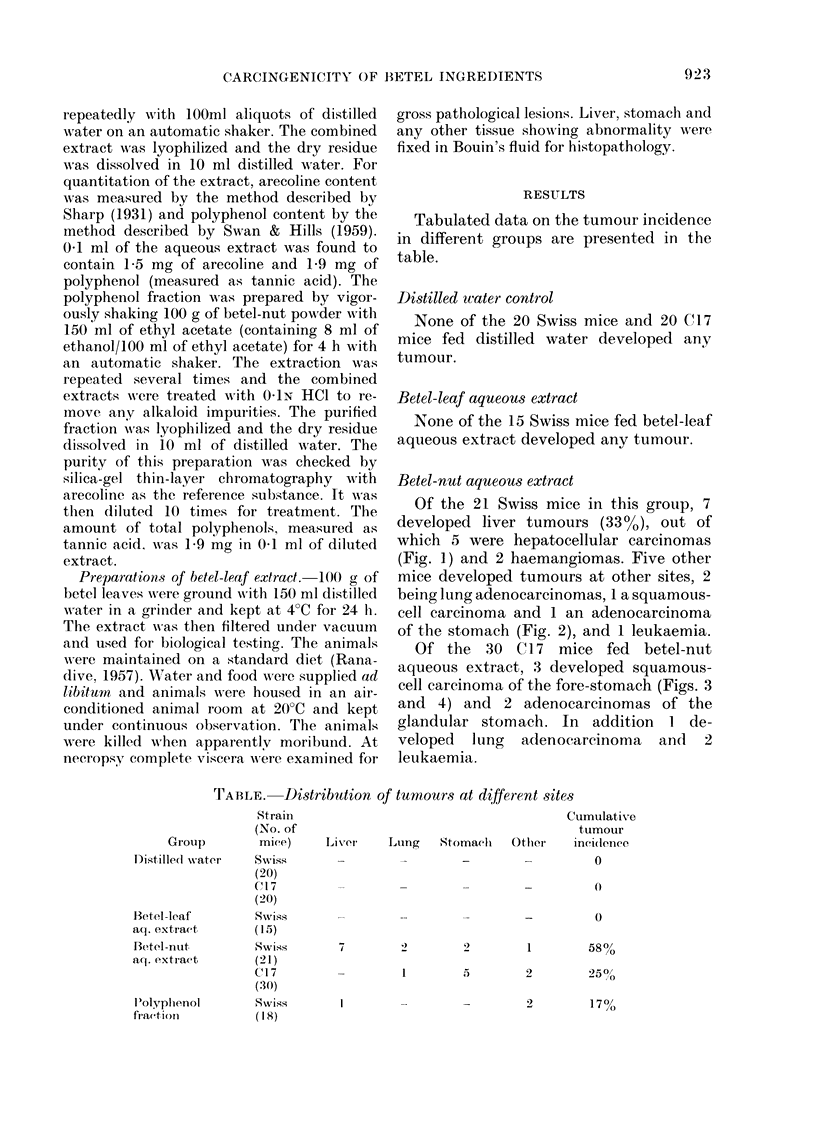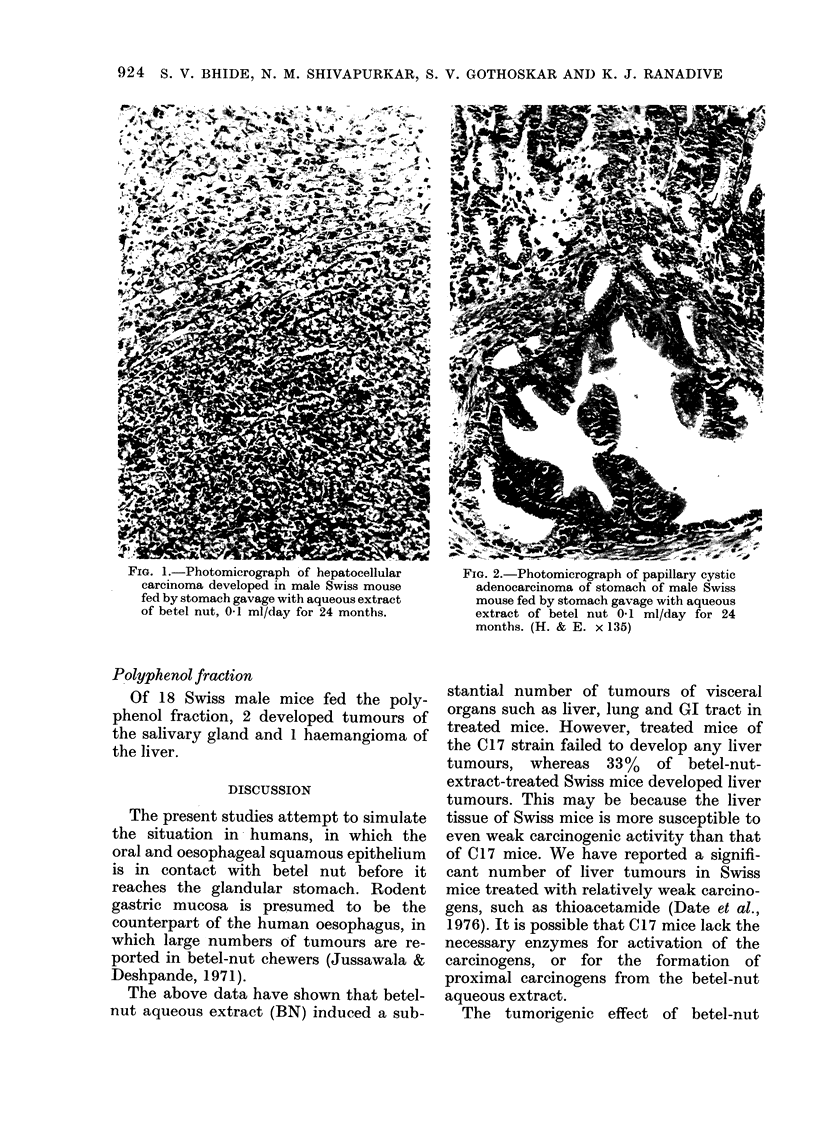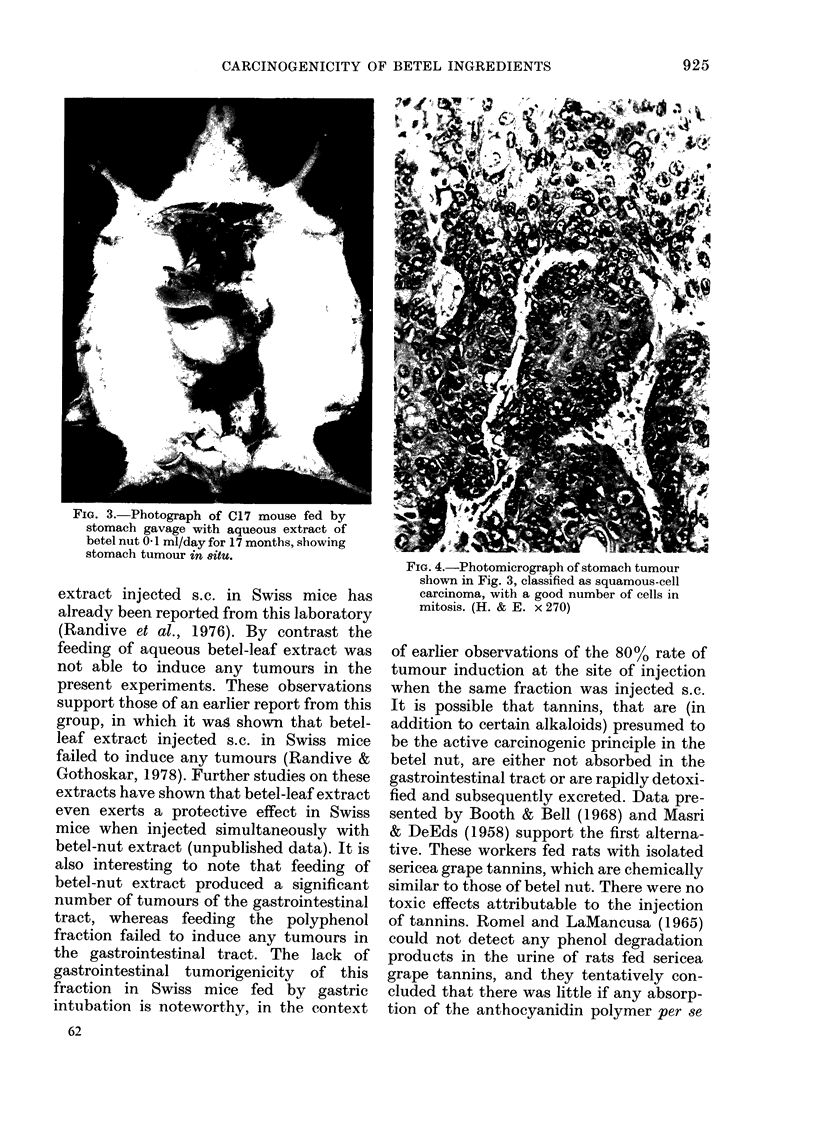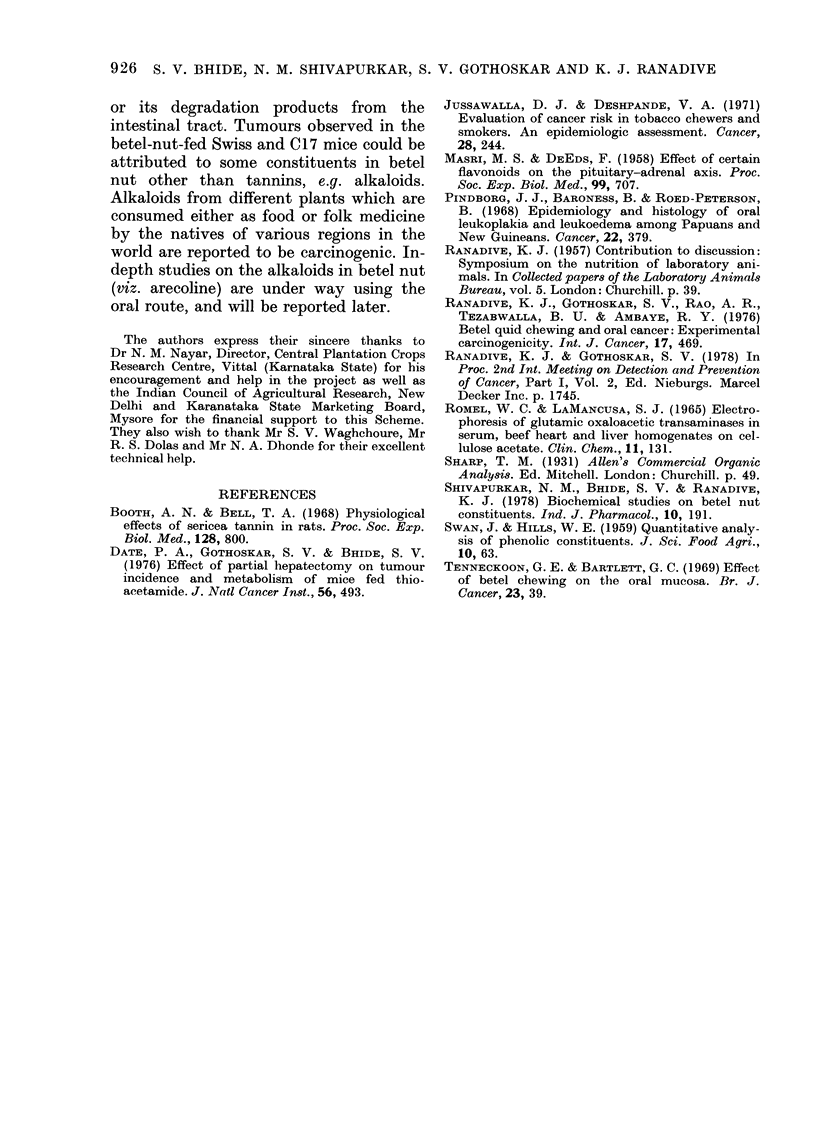# Carcinogenicity of betel quid ingredients: feeding mice with aqueous extract and the polyphenol fraction of betel nut.

**DOI:** 10.1038/bjc.1979.286

**Published:** 1979-12

**Authors:** S. V. Bhide, N. M. Shivapurkar, S. V. Gothoskar, K. J. Ranadive

## Abstract

**Images:**


*Br. J. Cancer* (1979) **40**, 922

### CARCINOGENICITY OF BETEL QUID INGREDIENTS: FEEDING MICE WITH AQUEOUS EXTRACT AND THE POLYPHENOL FRACTION OF BETEL NUT

S. V. BHIDE, N. M. SHIVAPURKAR, S. V. GOTHOSKAR AND K. J. RANADIVE

*From the Biology Division, Cancer Research Institute, Parel, Bombay* 400 012, *India*

Received 17 April 1979 Accepted 10 August 1979

**Summary.—Male mice of inbred strains Swiss and C17 were fed daily 5 times a week by intragastric tube 0·1 ml of betel-nut aqueous extract, betel-leaf aqueous extract and the polyphenol fraction of betel nut. Male mice of corresponding strains fed 0·1 ml of distilled water served as controls. Treated and control mice were kept under observation and killed when moribund. Betel-nut aqueous extract induced tumours of the gastrointestinal tract in 58% Swiss mice and 25% C17 mice. The polyphenol fraction by the same route induced tumours at other sites in 17% of the mice. Betel-leaf aqueous extract failed to induce any tumour in the treated mice, which supports an earlier report of the lack of any carcinogenic principle in betel leaf, an essential constituent of betel quid. Results are discussed in relation to the relevant literature.**

IN INDIA and the Far East the habit of betel chewing is a major factor in the cause of oral cancer. Many attempts have been made to develop a theory of the origin of betel-chewer's cancer, based on the chemical constituents of the chew. The chew or betel quid consists primarily of a few pieces of areca nut wrapped in the leaf of the betel vine, together with some lime. According to Tenneckoon & Bartlett (1969), lime might have an irritant action, but was used in such small quantities that dilution by saliva rendered it innocuous. In some localities certain other ingredients such as catechu and tobacco may be added, but they are not essential constituents of betel quid. In any case, similar pathological changes have been found in the absence of these ingredients (Pindborg *et al.*, 1968). Our preliminary studies on the aqueous extract of betel nut and its polyphenol fraction have shown that both produce a high percentage of fibrosarcomas at the site of injection in Swiss mice. Betel-leaf aqueous extract by s.c. injection, however, failed to produce any tumours (Ranadive *et al.*, 1976; Ranadive & Gothoskar, 1978). To simulate human conditions more closely we have tested betel-leaf aqueous extract, betel-nut aqueous extract and polyphenol extract of betel nut by gavage feeding.

### MATERIALS AND METHODS

Male mice of two inbred strains, Swiss and C17, were fed by intragastric tube 0·1 ml of aqueous extracts of betel nut and leaf, and also 0·1 ml of the polyphenol extract of betel nut, daily 5 times a week. Feeding was started at the age of 8–10 weeks and continued throughout the life-span of the treated animals. The following experimental groups were maintained.

1. Distilled water control group: 20 Swiss and 20 C17 mice.
2. Betel-leaf aqueous extract group: 15 Swiss mice.
3. Betel-nut aqueous extract group: 21 Swiss and 30 C17 mice.
4. Polyphenol fraction of betel nut group: 20 Swiss mice.

Betel-nut aqueous extract and the polyphenol extract (Shivapurkar *et al.*, 1978) were prepared as follows—

Cold aqueous extract of betel nut was prepared by shaking 100 g of betel-nut powder repeatedly with 100ml aliquots of distilled water on an automatic shaker. The combined extract was lyophilized and the dry residue was dissolved in 10 ml distilled water. For quantitation of the extract, arecoline content was measured by the method described by Sharp (1931) and polyphenol content by the method described by Swan & Hills (1959). 0·1 ml of the aqueous extract was found to contain 1·5 mg of arecoline and 1·9 mg of polyphenol (measured as tannic acid). The polyphenol fraction was prepared by vigorously shaking 100 g of betel-nut powder with 150 ml of ethyl acetate (containing 8 ml of ethanol/100 ml of ethyl acetate) for 4 h with an automatic shaker. The extraction was repeated several times and the combined extracts were treated with 0·1N HCl to remove any alkaloid impurities. The purified fraction was lyophilized and the dry residue dissolved in 10 ml of distilled water. The purity of this preparation was checked by silica-gel thin-layer chromatography with arecoline as the reference substance. It was then diluted 10 times for treatment. The amount of total polyphenols, measured as tannic acid, was 1·9 mg in 0·1 ml of diluted extract.

*Preparations of betel-leaf extract.*—100 g of betel leaves were ground with 150 ml distilled water in a grinder and kept at 4°C for 24 h. The extract was then filtered under vacuum and used for biological testing. The animals were maintained on a standard diet (Ranadive, 1957). Water and food were supplied *ad libitum* and animals were housed in an air-conditioned animal room at 20°C and kept under continuous observation. The animals were killed when apparently moribund. At necropsy complete viscera were examined for gross pathological lesions. Liver, stomach and any other tissue showing abnormality were fixed in Bouin's fluid for histopathology.

### RESULTS

Tabulated data on the tumour incidence in different groups are presented in the table.

*Distilled water control*

None of the 20 Swiss mice and 20 C17 mice fed distilled water developed any tumour.

*Betel-leaf aqueous extract*

None of the 15 Swiss mice fed betel-leaf aqueous extract developed any tumour.

*Betel-nut aqueous extract*

Of the 21 Swiss mice in this group, 7 developed liver tumours (33%), out of which 5 were hepatocellular carcinomas (Fig. 1) and 2 haemangiomas. Five other mice developed tumours at other sites, 2 being lung adenocarcinomas, 1 a squamous-cell carcinoma and 1 an adenocarcinoma of the stomach (Fig. 2), and 1 leukaemia.

Of the 30 C17 mice fed betel-nut aqueous extract, 3 developed squamous-cell carcinoma of the fore-stomach (Figs. 3 and 4) and 2 adenocarcinomas of the glandular stomach. In addition 1 developed lung adenocarcinoma and 2 leukaemia.

TABLE.—*Distribution of tumours at different sites*

| Group | Strain (No. of mice) | Liver | Lung | Stomach | Other | Cumulative tumour incidence |
|---|---|---|---|---|---|---|
| Distilled water | Swiss (20) | – | – | – | – | 0 |
| | C17 (20) | – | – | – | – | 0 |
| Betel-leaf aq. extract | Swiss (15) | – | – | – | – | 0 |
| Betel-nut aq. extract | Swiss (21) | 7 | 2 | 2 | 1 | 58% |
| | C17 (30) | – | 1 | 5 | 2 | 25% |
| Polyphenol fraction | Swiss (18) | 1 | – | – | 2 | 17% |

FIG. 1.—Photomicrograph of hepatocellular carcinoma developed in male Swiss mouse fed by stomach gavage with aqueous extract of betel nut, 0·1 ml/day for 24 months.

FIG. 2.—Photomicrograph of papillary cystic adenocarcinoma of stomach of male Swiss mouse fed by stomach gavage with aqueous extract of betel nut 0·1 ml/day for 24 months. (H. & E. ×135)

*Polyphenol fraction*

Of 18 Swiss male mice fed the polyphenol fraction, 2 developed tumours of the salivary gland and 1 haemangioma of the liver.

### DISCUSSION

The present studies attempt to simulate the situation in humans, in which the oral and oesophageal squamous epithelium is in contact with betel nut before it reaches the glandular stomach. Rodent gastric mucosa is presumed to be the counterpart of the human oesophagus, in which large numbers of tumours are reported in betel-nut chewers (Jussawala & Deshpande, 1971).

The above data have shown that betel-nut aqueous extract (BN) induced a substantial number of tumours of visceral organs such as liver, lung and GI tract in treated mice. However, treated mice of the C17 strain failed to develop any liver tumours, whereas 33% of betel-nut-extract-treated Swiss mice developed liver tumours. This may be because the liver tissue of Swiss mice is more susceptible to even weak carcinogenic activity than that of C17 mice. We have reported a significant number of liver tumours in Swiss mice treated with relatively weak carcinogens, such as thioacetamide (Date *et al.*, 1976). It is possible that C17 mice lack the necessary enzymes for activation of the carcinogens, or for the formation of proximal carcinogens from the betel-nut aqueous extract.

The tumorigenic effect of betel-nut

FIG. 3.—Photograph of C17 mouse fed by stomach gavage with aqueous extract of betel nut 0·1 ml/day for 17 months, showing stomach tumour *in situ.*

FIG. 4.—Photomicrograph of stomach tumour shown in Fig. 3, classified as squamous-cell carcinoma, with a good number of cells in mitosis. (H. & E. ×270)

extract injected s.c. in Swiss mice has already been reported from this laboratory (Randive *et al.*, 1976). By contrast the feeding of aqueous betel-leaf extract was not able to induce any tumours in the present experiments. These observations support those of an earlier report from this group, in which it was shown that betel-leaf extract injected s.c. in Swiss mice failed to induce any tumours (Randive & Gothoskar, 1978). Further studies on these extracts have shown that betel-leaf extract even exerts a protective effect in Swiss mice when injected simultaneously with betel-nut extract (unpublished data). It is also interesting to note that feeding of betel-nut extract produced a significant number of tumours of the gastrointestinal tract, whereas feeding the polyphenol fraction failed to induce any tumours in the gastrointestinal tract. The lack of gastrointestinal tumorigenicity of this fraction in Swiss mice fed by gastric intubation is noteworthy, in the context of earlier observations of the 80% rate of tumour induction at the site of injection when the same fraction was injected s.c. It is possible that tannins, that are (in addition to certain alkaloids) presumed to be the active carcinogenic principle in the betel nut, are either not absorbed in the gastrointestinal tract or are rapidly detoxified and subsequently excreted. Data presented by Booth & Bell (1968) and Masri & DeEds (1958) support the first alternative. These workers fed rats with isolated sericea grape tannins, which are chemically similar to those of betel nut. There were no toxic effects attributable to the injection of tannins. Romel and LaMancusa (1965) could not detect any phenol degradation products in the urine of rats fed sericea grape tannins, and they tentatively concluded that there was little if any absorption of the anthocyanidin polymer *per se*

or its degradation products from the intestinal tract. Tumours observed in the betel-nut-fed Swiss and C17 mice could be attributed to some constituents in betel nut other than tannins, *e.g.* alkaloids. Alkaloids from different plants which are consumed either as food or folk medicine by the natives of various regions in the world are reported to be carcinogenic. In-depth studies on the alkaloids in betel nut (*viz.* arecoline) are under way using the oral route, and will be reported later.

The authors express their sincere thanks to Dr N. M. Nayar, Director, Central Plantation Crops Research Centre, Vittal (Karnataka State) for his encouragement and help in the project as well as the Indian Council of Agricultural Research, New Delhi and Karanataka State Marketing Board, Mysore for the financial support to this Scheme. They also wish to thank Mr S. V. Waghchoure, Mr R. S. Dolas and Mr N. A. Dhonde for their excellent technical help.

### REFERENCES

BOOTH, A. N. & BELL, T. A. (1968) Physiological effects of sericea tannin in rats. *Proc. Soc. Exp. Biol. Med.*, **128**, 800.

DATE, P. A., GOTHOSKAR, S. V. & BHIDE, S. V. (1976) Effect of partial hepatectomy on tumour incidence and metabolism of mice fed thioacetamide. *J. Natl Cancer Inst.*, **56**, 493.

JUSSAWALLA, D. J. & DESHPANDE, V. A. (1971) Evaluation of cancer risk in tobacco chewers and smokers. An epidemiologic assessment. *Cancer*, **28**, 244.

MASRI, M. S. & DEEDS, F. (1958) Effect of certain flavonoids on the pituitary–adrenal axis. *Proc. Soc. Exp. Biol. Med.*, **99**, 707.

PINDBORG, J. J., BARONESS, B. & ROED-PETERSON, B. (1968) Epidemiology and histology of oral leukoplakia and leukoedema among Papuans and New Guineans. *Cancer*, **22**, 379.

RANADIVE, K. J. (1957) Contribution to discussion: Symposium on the nutrition of laboratory animals. In *Collected papers of the Laboratory Animals Bureau*, vol. 5. London: Churchill. p. 39.

RANADIVE, K. J., GOTHOSKAR, S. V., RAO, A. R., TEZABWALLA, B. U. & AMBAYE, R. Y. (1976) Betel quid chewing and oral cancer: Experimental carcinogenicity. *Int. J. Cancer*, **17**, 469.

RANADIVE, K. J. & GOTHOSKAR, S. V. (1978) In *Proc. 2nd Int. Meeting on Detection and Prevention of Cancer*, Part I, Vol. 2, Ed. Nieburgs. Marcel Decker Inc. p. 1745.

ROMEL, W. C. & LAMANCUSA, S. J. (1965) Electrophoresis of glutamic oxaloacetic transaminases in serum, beef heart and liver homogenates on cellulose acetate. *Clin. Chem.*, **11**, 131.

SHARP, T. M. (1931) *Allen's Commercial Organic Analysis*. Ed. Mitchell. London: Churchill. p. 49.

SHIVAPURKAR, N. M., BHIDE, S. V. & RANADIVE, K. J. (1978) Biochemical studies on betel nut constituents. *Ind. J. Pharmacol.*, **10**, 191.

SWAN, J. & HILLS, W. E. (1959) Quantitative analysis of phenolic constituents. *J. Sci. Food Agri.*, **10**, 63.

TENNECKOON, G. E. & BARTLETT, G. C. (1969) Effect of betel chewing on the oral mucosa. *Br. J. Cancer*, **23**, 39.